# The Dual Immunoregulatory function of *Nlrp12* in T Cell-Mediated Immune Response: Lessons from Experimental Autoimmune Encephalomyelitis

**DOI:** 10.3390/cells7090119

**Published:** 2018-08-27

**Authors:** Marjan Gharagozloo, Shaimaa Mahmoud, Camille Simard, Tara M. Mahvelati, Abdelaziz Amrani, Denis Gris

**Affiliations:** Program of Immunology, Department of Pediatrics, CR-CHUS, Faculty of Medicine and Health Sciences, University of Sherbrooke, Sherbrooke, QC J1H 5N4, Canada; Marjan.Gharagozloo@usherbrooke.ca (M.G.); Shaimaa.Mahmoud@usherbrooke.ca (S.M.); Camille.Simard@usherbrooke.ca (C.S.); t.mahvelati@gmail.com (T.M.M.); Abdelaziz.Amrani@usherbrooke.ca (A.A.)

**Keywords:** *Nlrp12*, CNS, inflammation, T cell, EAE, spontaneous EAE, TCR signaling, 2D2

## Abstract

Although the etiology of multiple sclerosis (MS) remains enigmatic, the role of T cells is unquestionably central in this pathology. Immune cells respond to pathogens and danger signals via pattern-recognition receptors (PRR). Several reports implicate *Nlrp12*, an intracellular PRR, in the development of a mouse MS-like disease, called Experimental Autoimmune Encephalomyelitis (EAE). In this study, we used induced and spontaneous models of EAE, as well as in vitro T cell assays, to test the hypothesis that *Nlrp12* inhibits Th1 response and prevents T-cell mediated autoimmunity. We found that *Nlrp12* plays a protective role in induced EAE by reducing IFNγ/IL-4 ratio in lymph nodes, whereas it potentiates the development of spontaneous EAE (spEAE) in 2D2 T cell receptor (TCR) transgenic mice. Looking into the mechanism of *Nlrp12* activity in T cell response, we found that it inhibits T cell proliferation and suppresses Th1 response by reducing IFNγ and IL-2 production. Following TCR activation, *Nlrp12* inhibits Akt and NF-κB phosphorylation, while it has no effect on S6 phosphorylation in the mTOR pathway. In conclusion, we propose a model that can explain the dual immunoregulatory function of *Nlrp12* in EAE. We also propose a model explaining the molecular mechanism of *Nlrp12*-dependent regulation of T cell response.

## 1. Introduction

Multiple sclerosis (MS) is a chronic autoimmune disease of the central nervous system (CNS), where autoreactive immune responses are involved in demyelination and CNS damage. The etiology and pathogenesis of the disease remain elusive. However, several lines of evidence demonstrate that adaptive immune response plays a key role in the pathogenesis of MS and experimental autoimmune encephalomyelitis (EAE), the mouse model of MS [1,2,3]. The major components of the adaptive immunity, T cells, are initially activated by antigen presenting cells (APCs) in lymph nodes. Activated T cells migrate into the CNS across the blood brain barrier (BBB) and reactivated again in perivascular space, where CNS-resident cells including microglia and macrophages present myelin antigens to T cells [4]. Thus, those activated CD4^+^ T cells orchestrate the functions of other adaptive immune cells, such as CD8^+^ T cells and B cells, as well as innate immune cells in the CNS and periphery [5]. Depending on the composition of the cytokine milieu, naïve CD4^+^ T cells may differentiate into different T helper (Th) subsets including Th1, Th2 and Th17 that produce signature cytokines such as IFNγ, IL-4 and IL-17 respectively. The differentiation of naïve CD4^+^ T cells into Th1, Th2, or Th17 types are governed by transcription factors, known as Tbet, GATA3 and RORγt respectively [6]. Th subsets affect the CNS inflammation in different ways. Th1 and Th17 responses potentiate the CNS inflammation, while Th2 response dampens inflammatory response and protects CNS damage [6]. These findings highlight the importance of T cell-mediated immunity in MS pathology. 

APCs including dendritic cells, macrophages and microglia are innate immune cells that trigger T cell activation [7]. These cells create the first line of response by recognizing pathogens and/or danger signals via pattern-recognition receptors (PRR) [8]. NOD-like receptors (NLRs) are intracellular PRR that are mainly expressed by cells of hematopoietic origin and regulate both innate and adaptive immune responses [9]. Recently, NLRs have gained more attention since 3 members of the family including CIITA, Nlrc5 and Nlrp3 regulate transcription of molecules that shape adaptive immune responses. CIITA [10] and Nlrc5 [11,12] show transcriptional activities for MHC II and MHC I molecules respectively, while *Nlrp3* acts as a Th2 transcription factor and promotes IL-4 production [13]. In addition, activation of NLRs often leads to the production and secretion of pro–inflammatory cytokines such as IL-1β and IL-18 that in turn potentiate differentiation of Th1 and Th17 subsets [9,14]. These findings highlight the key role of NLR proteins in shaping T cell response and adaptive immunity. 

Not all NLRs are pro–inflammatory. *Nlrp12* is a recently discovered member of NLRs that is shown to be a negative regulator of both canonical and non-canonical nuclear factor-κB (NF-κB) signaling pathways [15]. Previous studies showed that Nlrp12*^−/−^* mice are highly vulnerable to inflammatory diseases such as experimental colitis and colorectal tumor development [16,17,18,19]. In the context of CNS inflammation, the lack of *Nlrp12* resulted in increased CNS inflammation and exacerbated course of EAE [19]. *Nlrp12^−/−^* mice developed earlier and more severe form of EAE than wild-type (WT) mice. This phenotype parallel with significant increases in the expression of pro-inflammatory genes in the spinal cords of *Nlrp12^−/−^* mice relative to WT mice. Experiments using mouse primary microglia cultures demonstrated that *Nlrp12* significantly inhibits production of the inflammatory mediators such as *inducible* nitric oxide synthase (iNOS), Tumor Necrosis Factor (TNF)α, IL-6 and nitric oxide (NO) [19]. However, the ability of *Nlrp12* to modulate T cell responses remains poorly defined. 

A recent article by Lukens et al. revealed that *Nlrp12* is expressed not only by myeloid cells but also by T cells. It negatively regulates NF-κB signaling, T cells proliferation and the secretion of Th1/Th2/Th17 cytokines [20]. Non-surprisingly, *Nlrp12* deficient mice developed enhanced inflammatory symptoms in T-cell-mediated autoimmune diseases such as colitis and atopic dermatitis [20]. However, in EAE model, lack of *Nlrp12* promotes Th2 response and IL-4 secretion, which results in a milder form of EAE with atypical symptoms, including ataxia and impaired balance control [20]. Collectively, current findings and controversies indicate that the exact immunoregulatory functions of *Nlrp12* in T cell activation and T cell-mediated autoimmunity are poorly understood. 

In this study, we investigated the immunoregulatory role of *Nlrp12* in T cell responses using classical induced-EAE and spontaneous EAE (spEAE) models. We further characterized the role of *Nlrp12* in regulating T cell receptor (TCR) signaling pathways and IL-2 production. 

## 2. Materials and methods

### 2.1. Mice

All the protocols and procedures were approved by the University of Sherbrooke Animal Facility and Use Committee (Protocols #280-15, 4 April 2017; #335-17B, 22 February 2018). *Nlrp12* knock-out *(Nlrp12^−/−^)* mice on C57BL/6J background were kindly provided by Dr. Jenny P.Y. Ting (Chapel Hill, NC, USA). Mice were backcrossed for at least 15 generation. The 2D2 transgenic mice expressing a TCR specific for the myelin oligodendrocyte (MOG_35__–__55_) peptide were purchased from Jackson Laboratory. *Nlrp12^−/−^* and WT mice were crossed with 2D2 mice to generate *Nlrp12^−/−^* 2D2 mice. We genotyped all the animals for *Nlrp12* and 2D2 (Supplementary protocol) and only those animals that were *Nlrp12^−/−^* and 2D2^+^ were included in the study (Supplementary Figure S1). Moreover, the expression of Vβ11 receptor was verified with flow cytometry. The mice were maintained under specific pathogen-free conditions in the animal facility of the faculty of medicine, at the University of Sherbrooke. 

### 2.2. Induction of EAE and Tissue Collection

EAE was induced in 8–10-week old WT or *Nlrp12^−/−^* female mice as previously described [19]. An emulsion mixture of MOG_35−55_ (Genemed Synthesis Inc., San Antonio, TX, USA), complete Freund’s Adjuvant (CFA) (Sigma-Aldrich, St. Louis, MO, USA) and *Mycobacterium tuberculosis* H37 RA (Difco Laboratories, Detroit, MI, USA) was prepared and injected subcutaneously in the flank with a total of 200 μg MOG_35–55_ and 500 μg *Mycobacterium*. Mice were also injected intraperitoneally on days 0 and 2 with 200 ng Pertussis toxin (List Biological Laboratories Inc., Campbell, CA, USA). After 3 weeks of immunization, mice were sacrificed, perfused with ice-cold phosphate-buffered saline (PBS) (Wisent, St. Bruno, QC, Canada) and the CNS tissues were collected.

### 2.3. Intracellular Staining and Flow Cytometry

CD4^+^ T cells were purified from lymph nodes and spleens using Mouse CD4^+^ T Cell Isolation Kit (eBioscience, San Diego, CA, USA) and activated with plate-bound anti-CD3 (eBioscience, clone:145-2C11, 1 μg/mL) and anti-CD28 (eBioscience, clone: 37.51, 2 μg/mL) antibodies for indicated times. T cell proliferation was assessed by Ki67 intranuclear staining following fixation and permeabilization in the Foxp3/Transcription Factor staining kit (eBioscience). For intracellular staining of cytokines, the cells were stimulated with phorbol 12-myristate 13-acetate (PMA; 500 ng/mL, Sigma Chemical Co., St. Louis, MO, USA) and ionomycin (1 μg/mL, Calbiochem Corp., La Jolla, CA, USA) for 5 h at 37 °C in the presence of Brefeldin A (10 μg/mL, eBioscience). Cells were stained with anti-CD4-FITC antibody (eBioscience), fixed, permeabilized and then stained with anti-IFNγ-PE, anti-IL-4-PE, IL-17-PerCP-Cy5.5, Tbet-PE, or RORγt-PE antibody, as per the manufacturer’s instructions (eBioscience). Sample acquisition was performed with Beckman Coulter CytoFlex (Beckman Coulter, Brea, CA, USA) and data were analyzed using CytExpert 2 software (Beckman Coulter, Brea, CA, USA). Plots were prepared using CytExpert 2 and FlowJo (San Carlos, CA, USA) software.

### 2.4. Quantitative RT-PCR 

RNA was extracted from CD4^+^ T cells using TRIzol reagent (Life Technologies Inc., Burlington, ON, USA) and cDNA was synthesized as previously described [19]. Reverse transcription PCR (RT-PCR) was used to verify the expression of *Nlrp12* in activated T cell using KiCqStart™ SYBR^®^ Green qPCR ReadyMix (Sigma Aldrich, St. Louis, MO, USA). Primers (IDT, Coralville, IA, USA) sequences were as follows: *Nlrp12F*: 5′-CCT CTT TGA GCC AGA CGA AG-3′, *Nlrp12R*: 5′-GCC CAG TCC AAC ATC ACT TT-3′, *18SF*: 5′-CGG CTA CCA CAT CCA AGG AA-3′ and *18SR*: 5′-GCT GGA ATT ACC GCG GCT-3′. The relative expression was calculated using the ΔΔC_T_ method [21].

### 2.5. Cytokine Measurement

IFNγ and IL-4 cytokines in the supernatant of activated CD4^+^ T cell culture and tissue lysates were measured using ELISA kits as previously described [19]. Briefly, lymph node, spinal cord and cerebellum tissues were homogenized in lysis buffer supplemented with protease inhibitors (Cell Signaling Technology, Danvers, MA, USA) by rapid agitation using 3-mm stainless beads and a TissueLyser II (Qiagen, Hilden, Germany) homogenizer for 2 min. The levels of IL-4 in tissue lysates were determined using a high sensitivity IL-4 ELISA Kit (eBioscience, San Diego, CA, USA) according to the manufacturer’s instruction. The amount of IFNγ was determined using IFNγ kit purchased from PeproTech (Rocky Hill, NJ, USA).

### 2.6. Differentiation of 2D2 T Cells Toward Th1 or Th17 In Vitro

Naïve CD4^+^ T cells were purified from the spleens and lymph nodes of *Nlrp12^−/−^* 2D2 and WT 2D2 mice using MagniSort Naïve CD4 T Cell Enrichment Kit (eBiosciences). Purified CD4^+^ T cells were stimulated with MOG (50 µg/mL) in the presence of WT splenocytes at 1:1 ratio and Th1-, Th2- or Th17- polarizing condition (Th1: IL-12 (10 ng/mL), anti-IL-4 (10 μg/mL), IL-2 (10 ng/mL); Th2: IL-4 (10 μg/mL), hTGF-β1 (10 ng/mL), IL-2 (10 ng/mL) and Th17: anti-IL-12 (10 μg/mL), anti-IL-4 (10 μg/mL), anti-IFN-γ (10 μg/mL), mIL-6 (10 ng/mL), hTGF-β1 (10 ng/mL)). Recombinant cytokines and antibodies were purchased from Biolegend (San Diego, CA, USA) and eBioscience (San Diego, CA, USA), respectively. After 72 h of culture, cells were stained for MOG-TCR transgenic surface marker, Vβ11 and Th1- or Th17- associated markers (intracellular cytokine and transcription factor). The percentage of Th1 or Th17 cells were evaluated by Flow cytometry.

### 2.7. T Cell Activation and Signaling Pathways 

To analyze p65 phosphorylation upon CD3 cross-linking, CD4^+^ T cells were purified from the lymph nodes of *Nlrp12^−/−^* and WT mice and incubated with 10 μg/mL anti-CD3 (eBioscience, San Diego, CA, USA) for 30 min on ice, followed by washing and re-suspending the cells in serum-free medium. T cells were incubated with 10 μg/mL cross-linking anti-mouse IgG antibody (R&D systems, Minneapolis, MN, USA) for 20 min at 37 °C. Akt phosphorylation was quantified after activation of CD4^+^ T cells with PMA/Iono for 10 min at 37 °C, while the phosphorylation of S6 was analyzed after 24 h activation of CD4^+^ T cells with plate-bound anti-CD3/CD28 antibodies. Following stimulation in a defined period of time, T cells were washed and lysed in the lysis buffer plus proteinase and phosphatase inhibitor (Cell Signaling Technology). Proteins were then separated by on 10% SDS-polyacrylamide gels and transferred to nitrocellulose membrane. Transfers were blocked for 1h at room temperature with 5% nonfat milk in TBS/0.1% Tween 20 (TBST) and then incubated overnight at 4 °C in the primary antibodies (Cell Signaling Technology) diluted in TBST. The membranes were washed 3 times with TBST and incubated in horseradish peroxidase (HRP)-conjugated goat anti-rabbit antibody (Cell Signaling Technology) diluted 1/1000 in TBST for 1 h at room temperature. The immunoblots were developed with Lumigen ECL ultra reagent (Lumigen, Southfield, MI, USA), imaged with ChemiDoc^TM^ (Bio-Rad, Hercules, CA, USA) and analyzed using Image Lab software (Bio-Rad). 

### 2.8. Phosphoflow Cytometry

Phosphorylation of S6 ribosomal protein was confirmed by flow cytometry. Following 24 h stimulation of CD4^+^ T cells with plate-bound anti-CD3/CD28, cells were immediately fixed and permeabilized using Fix and Perm Kit (eBioscience). After washing with perm buffer (eBioscience) and blocking with 5% Fetal Bovine Serum, cells were incubated with Phospho-S6 antibody (Cell Signaling Technology) for 1h at room temperature, followed by washing with perm buffer and incubation with Alexa Fluor^®^ 555 (Cell Signaling Technology) conjugated antibody. Samples were acquired by Beckman Coulter CytoFlex and data was analyzed using CytExpert 2 software (Beckman Coulter, Brea, CA, USA).

### 2.9. SpEAE and Histological Analysis 

WT 2D2 or *Nlrp12^−/−^* 2D2 mice were monitored for the development of spEAE. Animals were sacrificed after 4 months of monitoring or after the development of EAE at the peak score of 4. The immunized mice were sacrificed as described in Section 2.2 and the spinal cords were removed and fixed in 4% formaldehyde for 24 h. The spinal cord tissues were embedded in paraffin and cut into 5-μm sections and stained with hematoxylin and eosin (H&E) stain and immunofluorescence for astrocyte marker (GFAP), microglia marker (Iba1) and myelin basic protein (MBP). All slides were scanned using a digital slide scanner NanoZoomer-XR C12000 (Hamamatsu Photonics, Hamamatsu, Japan) and viewed using NDPview2 software (Hamamatsu Photonics, Hamamatsu, Japan).

### 2.10. Analysis of CNS-Infiltrating Mononuclear Cells by Flow Cytometry

Dissected spinal cords were filtered through a 70 mm nylon sieve (BD Pharmingen, Becton Dickinson, Franklin Lakes, NJ, USA) with a syringe plunger to get a uniform tissue homogenate that was digested with collagenase D (2.5 mg/mL, Roche Diagnostics, Risch-Rotkreuz, Switzerland) and DNase I (1 mg/mL, Sigma-Aldrich) with agitation at 37 °C for 45 min. Mononuclear cells were isolated by percoll (Sigma-Aldrich) centrifugation, in which 300 µL percoll was mixed with 1 mL cell suspension overlaid with 700 µL Hank’s Balanced Salt Solution (HBSS). The samples were centrifuged at 12,000× *g* for 15 min without break. Myelin and cell debris were aspirated and the mononuclear pellet was re-suspended and washed with HBSS before staining for surface markers and analysis by flow cytometry.

### 2.11. Statistical Analysis

All statistical analyses were conducted using GraphPad Prism 7 software (GraphPad, San Diego, CA, USA). Results were expressed as the mean ± standard deviation. Statistical differences between WT and *Nlrp12^−/−^* samples were assessed by Student’s *t*-test. Level of *Nlrp12* expression was assessed by one-way ANOVA and cellular signaling by two-way ANOVA. The significance level was set at *p* < 0.05.

## 3. Results

### 3.1. Nlrp12 Modulates Th1/Th2 Balance in EAE Mice 

**Figure 1:**
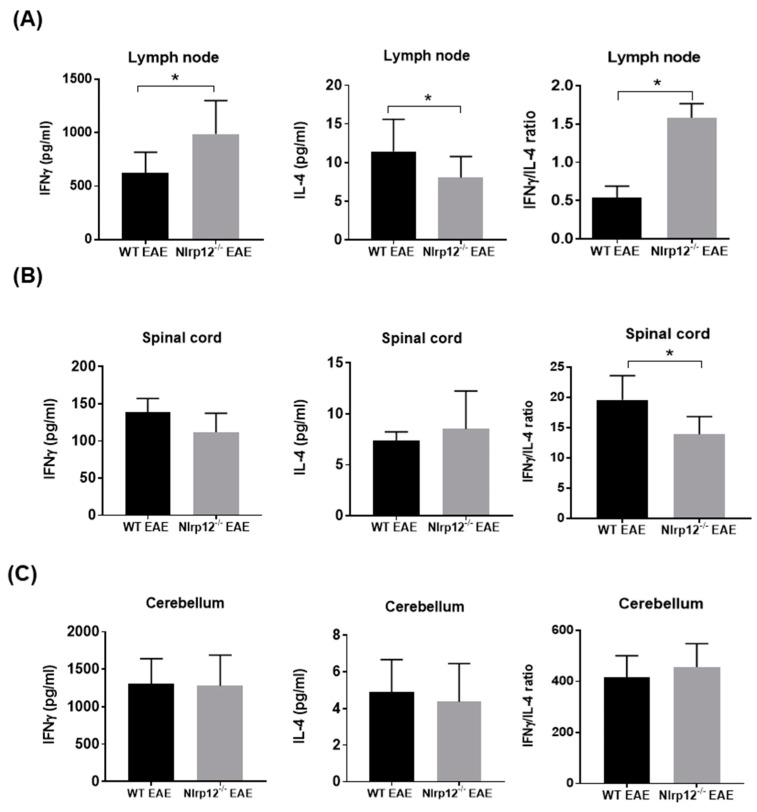
*Nlrp12* inhibits Th1 response in Experimental Autoimmune Encephalomyelitis (EAE). Th1- and Th2- related cytokines in tissue lysates from EAE mice. Central nervous system (CNS) tissues and lymph nodes were collected from EAE mice after 3 weeks of immunization with myelin oligodendrocyte/complete Freund’s adjuvant (MOG/CFA). The tissues were homogenized in lysis buffer and cytokine levels were measured in tissue lysate by ELISA. (**A**) The levels of IFNγ, IL-4 and IFNγ/IL-4 ratio in lymph node extracts from WT and *Nlrp12^−/−^* EAE mice; (**B**) The levels of IFNγ, IL-4 and their ratios in the spinal cord and (**C**) cerebellum of EAE mice, *n* = 5–6 per group, * *p* < 0.05.

Our previous study demonstrated that *Nlrp12^−/−^* mice develop earlier and more severe EAE compared to WT mice (Supplementary Figure S2) [19]. Since Th1 cells play a crucial role in the pathogenesis of EAE, we examined whether the imbalance of Th1/Th2 response might lead to exacerbated EAE in *Nlrp12^−/−^* mice. We collected lymph nodes and the CNS tissues including spinal cord and cerebellum from WT and *Nlrp12^−/−^* EAE mice at day 21 after MOG-CFA immunization and measured the level of IFNγ and IL-4 as the designated cytokines for Th1 and Th2 cells respectively. As shown in Figure 1, we found significantly higher levels of IFNγ in lymph node extracts from *Nlrp12^−/−^* EAE mice, while IL-4 levels were significantly lower than WT EAE mice. In addition, the ratio of IFNγ/IL-4 was higher in lymph node extracts of *Nlrp12^−/−^* EAE mice, suggesting a higher Th1/Th2 ratio in *Nlrp12^−/−^* EAE (Figure 1A). No difference was observed in the levels of IFNγ and IL-4 in the extracts of CNS tissues (spinal cord and cerebellum) (Figures 1B and 1C), however, IFNγ/IL-4 ratio was significantly lower in spinal cords from *Nlrp12^−/−^* EAE mice compared to WT EAE mice (Figure 1B). We then evaluated the production of cytokines in ex vivo activated CD4^+^ T cells. 

### 3.2. Nlrp12 Inhibits the Production of IFNγ by CD4^+^ T Cells In Vitro

**Figure 2:**
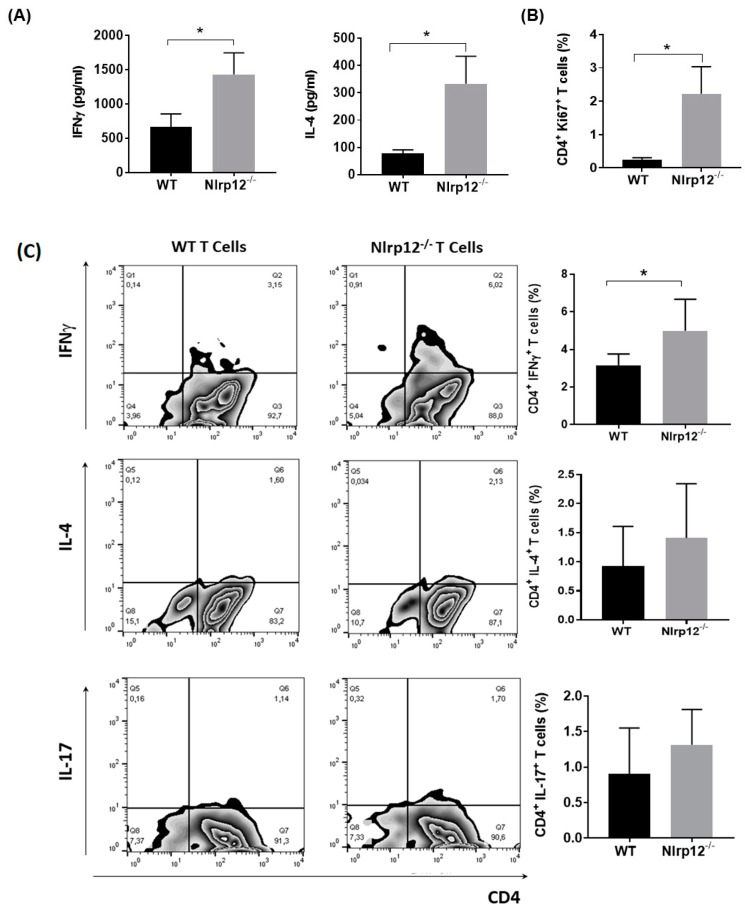
*Nlrp12* inhibits T cell proliferation and cytokine production by CD4^+^ T cells. (**A**) Purified CD4^+^ T cells from WT and *Nlrp12^−/−^* mice were stimulated with anti-CD3/CD28 for 72 h. Cell culture supernatants were collected and the levels of IFNγ and IL-4 were measured by ELISA, *n* = 4; (**B**) Purified CD4^+^ T cells from WT and *Nlrp12^−/−^* mice were stimulated with anti-CD3/CD28 for 24 h and the proliferation of CD4^+^ T cells was evaluated using Ki67 staining and flow cytometry; (**C**) CD4^+^ T cells were activated with anti-CD3/CD28 for 48 h and the expression of intracellular cytokines was measured using flow cytometry. Representative flow cytometric plots show the expression of IFNγ, IL-4 and IL-17 in CD4^+^ T cells, *n* = 4; * *p* < 0.05.

To determine whether *Nlrp12* could inhibit IFNγ production by T cells, we purified CD4^+^ T cells from WT and *Nlrp12^−/−^* mice and stimulated them with anti-CD3/CD28 antibodies to activate T cells with TCR and costimulatory signals simultaneously [22]. After 72 h, cell culture supernatants were collected and the levels of IFNγ and IL-4 were measured by ELISA. As shown in Figure 2A, activated CD4^+^ T cells from *Nlrp12^−/−^* mice secreted significantly higher levels of IFNγ and IL-4 compared to WT T cells. We then tested whether the increased levels of both Th1- and Th2-associated cytokines were related to the increased proliferation of *Nlrp12^−/−^* T cells compared to WT T cells. Ki67 is a nuclear protein that is expressed by the cells in all cell cycle phases except G0 phase [23]. Following activation by anti-CD3/CD28 antibody for 24 h, the CD4^+^ T cells were stained for Ki67 and analyzed by flow cytometry. Results showed significantly higher numbers of *Nlrp12^−/−^* T cells in the cell cycle compared to WT T cells (Figure 2B). Flow cytometric analysis of intracellular cytokines revealed that higher percentages of CD4^+^ T cells produced IFNγ in *Nlrp12^-/-^* activated T cells. However, no significant difference was detected between *Nlrp12^−/−^* and WT T cells in the percentage of IL-4 or IL-17 producing T cells after 3 days of activation with anti-CD3/CD28 antibodies in vitro (Figure 2C). We then examined whether the exacerbated EAE in *Nlrp12^−/−^* mice was associated with the increased differentiation of myelin-specific T cells to Th1 or Th17 cells.

### 3.3. Nlrp12 Has No Effect on T Cell Differentiation toward Th1 and Th17

**Figure 3:**
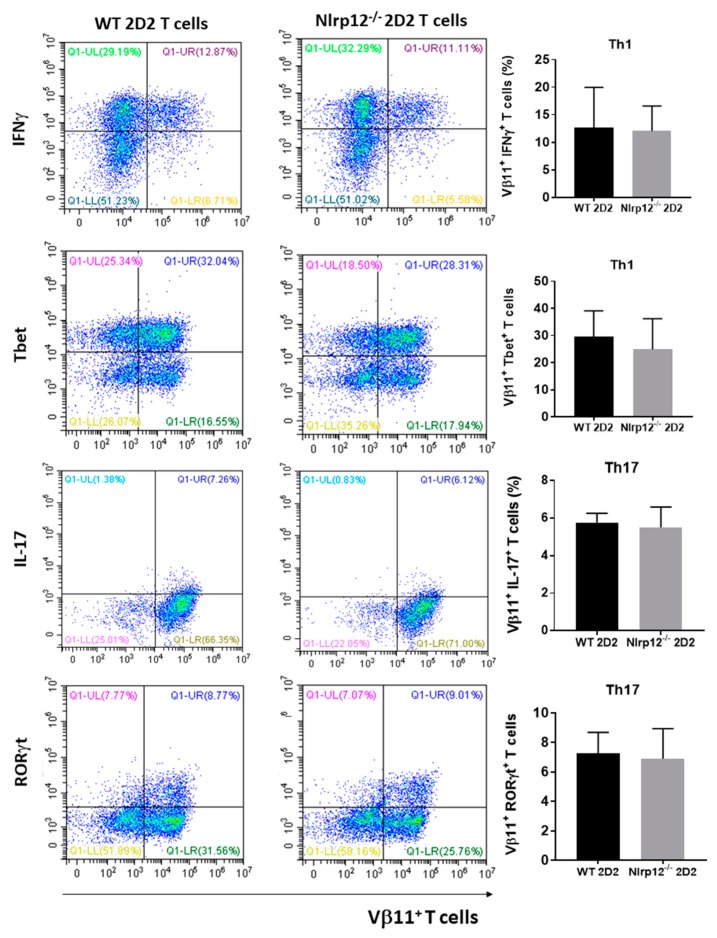
*Nlrp12* does not affect the differentiation of naïve CD4^+^ T cells to Th1 or Th17. Naïve CD4^+^ T cells from WT and *Nlrp12^−/−^* mice were purified and stimulated with MOG-splenocytes in the presence of Th1 or Th17 polarizing cytokines for 3 days. After 5 h stimulation with PMA/ionomycin, cells were stained for Vβ11 and Th1- or Th17- associated cytokine/transcription factor and analyzed by flow cytometry, *n* = 3; Representative flow cytometric plots show the expression of Th1- and Th17-related markers in Vβ11^+^ T cells.

We evaluated whether *Nlrp12* would favor differentiation of naïve T cells towards inflammatory Th subsets. We purified naïve CD4^+^ T cells from lymph nodes and spleens of *Nlrp12^−/−^* 2D2 and WT 2D2 mice and cultured with MOG-pulsed WT splenocytes in the presence or absence of Th1 or Th17 polarizing cytokines. After 72 h of incubation, cells were harvested and stained for CD4 surface marker and Th1 (IFNγ and Tbet) or Th17 (IL-17 and RORγt) intracellular markers. Flow cytometry results show that *Nlrp12* did not affect the differentiation of CD4^+^ T cells toward Th1 or Th17 (Figure 3). 

### 3.4. Nlrp12 Expression Is Increased in Activated T Cells 

**Figure 4:**
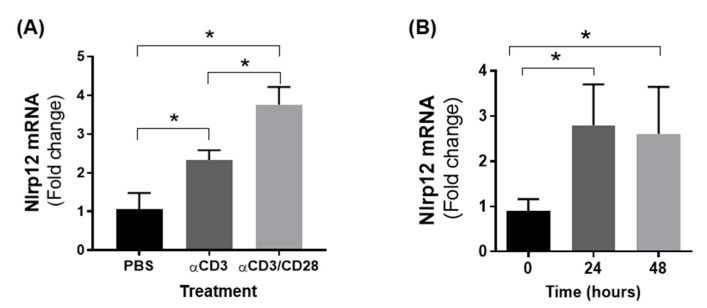
T cell activation increases *Nlrp12* mRNA expression. (**A**) Increased expression of *Nlrp12* mRNA in CD4^+^ T cells activated with anti-CD3 or anti-CD3/CD28 antibodies for 24 h. The higher expression was found in T cells activated with anti-CD3/CD28 antibody compared to T cell treatment with anti-CD3 or PBS; (**B**) The expression of *Nlrp12* is increased in activated CD4^+^ T cells after 24 and 48 h activation with anti(α)-CD3/CD28 antibody; results are presented relative to the expression of *Nlrp12* in inactive cells; *n* = 4, * *p* < 0.05.

To test whether TCR activation modulated the expression of *Nlrp12*, we activated CD4^+^ T cells with either anti-CD3 or anti-CD3/CD28 antibodies for 24 h and measured the expression of *Nlrp12* gene by qPCR. As shown in Figure 4A, the mRNA expression of *Nlrp12* was significantly increased in T cells following activation by anti-CD3 or anti-CD3/CD28 antibodies. The level of *Nlrp12* expression remains high even after 48 h stimulation of T cells by anti-CD3/CD28 (Figure 4B). Since we observed a change in *Nlrp12* expression soon after T cell activation, we asked the question whether *Nlrp12* could modify early TCR signaling events before commitment of T cells to a certain Th subset. 

### 3.5. Nlrp12 Inhibits Phosphorylation of Akt and NF-κB Signaling in Activated T Cells

**Figure 5:**
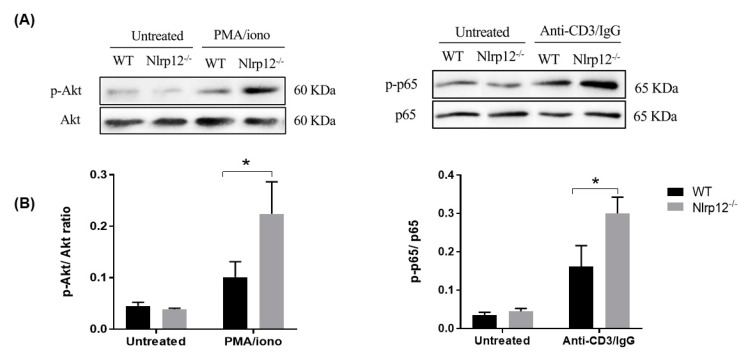
*Nlrp12* inhibits phosphorylation of Akt and p65 in CD4^+^ T cells. The cells were activated either with PMA/ionomycin for 10 min or with cross-linking anti-CD3 antibody (anti-CD3/IgG) for 20 min at 37 °C. (**A**) Samples were analyzed by SDS-PAGE and immunoblotting for phospho-Akt (S473) and phospho-p65. Total Akt and p65 expression in the same lysates were used to normalize the expression of phosphorylated molecule between samples; (**B**) The ratio of phosphorylated to total molecules were compared between WT and *Nlrp12^−/−^* cells, *n* = 3, * *p* < 0.05.

Akt can be activated by TCR signaling in T cell. Accordingly, we studied the early TCR signaling and Akt phosphorylation in T cells downstream of TCR, where Akt is activated by protein kinase C (PKC). For mitogenic stimulation that bypasses the TCR, T cells were treated with the combination of PMA, a permeable specific activator of PKC and calcium ionophore that synergizes with PMA effect in enhancing the activation of PKC [24]. We activated CD4^+^ T cells by PMA/ionomycin or CD3 cross-linking antibody and evaluated the phosphorylation of Akt and p65 NF-κB subunit by Western blot. As shown in Figure 5, a significant increase in the phosphorylation of Akt and p65 NF-κB subunit were found in *Nlrp12^−/−^* CD4^+^ T cells compared to WT CD4^+^ T cells. 

### 3.6. Nlrp12 Inhibits IL-2 Synthesis but Does Not Modify Ca^2+^/Calmodulin-Dependent T Cell Activation

**Figure 6:**
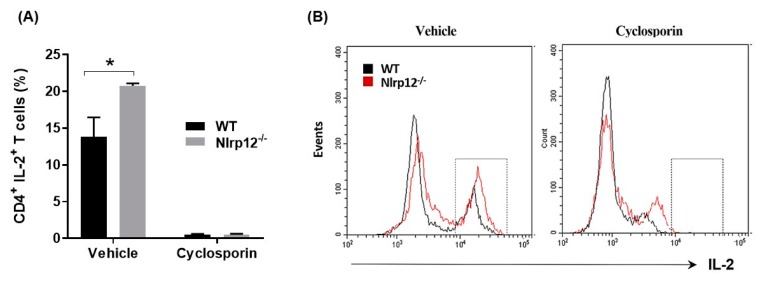
*Nlrp12* inhibits IL-2 synthesis by activated T cells. (**A**) CD4^+^ T cells from WT and *Nlrp12^−/−^* mice were activated with anti-CD3/CD28 for 24 h in the presence of cyclosporin or its vehicle (DMSO) for 24 h and intracellular expression of IL-2 were quantified using flow cytometry; (**B**) Representative flow cytometric histograms showing the population of IL-2^+^ T cells in vehicle or cyclosporin-treated group, *n* = 3, * *p* < 0.05.

Since the phosphorylation of NF-κB promotes IL-2 synthesis, we tested whether *Nlrp12* inhibit IL-2 production in MOG-specific transgenic CD4^+^ T cells. CD4^+^ T cells from *Nlrp12^−/−^* 2D2 and WT 2D2 mice were activated with MOG-pulsed splenocytes and IL-2 expression by CD4^+^ T cells was quantified using flow cytometry. Furthermore, to test whether *Nlrp12* modifies Ca^2+^/calmodulin-dependent T cell activation, we incubated the T cells with and Ca^2+^/calmodulin inhibitor (cyclosporin) for 24 h and quantified the percentage of CD4^+^ IL-2^+^ T cells by flow cytometry. As shown in Figure 6, *Nlrp12* inhibits IL-2 production by MOG-activated CD4^+^ T cells, however, it does not interfere with the immunosuppressive activities of cyclosporine, since cyclosporin inhibits IL-2 production in both *Nlrp12^−/−^* and WT T cells.

### 3.7. Nlrp12 Does Not Affect the Phosphorylation of S6 Ribosomal Protein in mTOR Pathway

**Figure 7:**
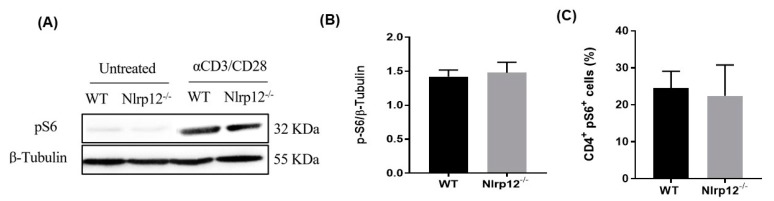
*Nlrp12* does not modify mTOR activity. (**A**) The phosphorylation of S6 ribosomal protein (pS6) as an indicator of mTOR activity was assessed by Western blotting; (**B**) The ratio of pS6 to β-Tubulin in WT and *Nlrp12^−/−^* CD4^+^ T cells activated with anti-CD3/CD28 for 24 h, as quantified by Western blot, *n* = 3; (**C**) Phospho-flow analysis of pS6 in CD4^+^ T cells activated with anti-CD3/CD28 for 24 h, *n* = 3.

IL-2 is known to activate mTOR pathway and to promote cellular growth and clonal expansion of effector T cells [25]. Since we found an increased production of IL-2 and enhanced proliferation of *Nlrp12^−/−^* CD4^+^ T cells, we investigate whether *Nlrp12* affects mTOR pathway in T cells. We evaluated the phosphorylation of S6 ribosomal protein, which is a critical component of 40 S ribosomal subunit and sits at the downstream of mTOR pathway. Following 24 h activation by plate-coated anti-CD3/CD28 antibodies, no significant difference was found between *Nlrp12^−/−^* and WT CD4^+^ T cells in the levels of phospho-S6, determined by Western blotting and flow cytometry (Figure 7A–C). 

### 3.8. Nlrp12^−/−^ 2D2 Mice Are Resistant to the Development of spEAE 

**Figure 8:**
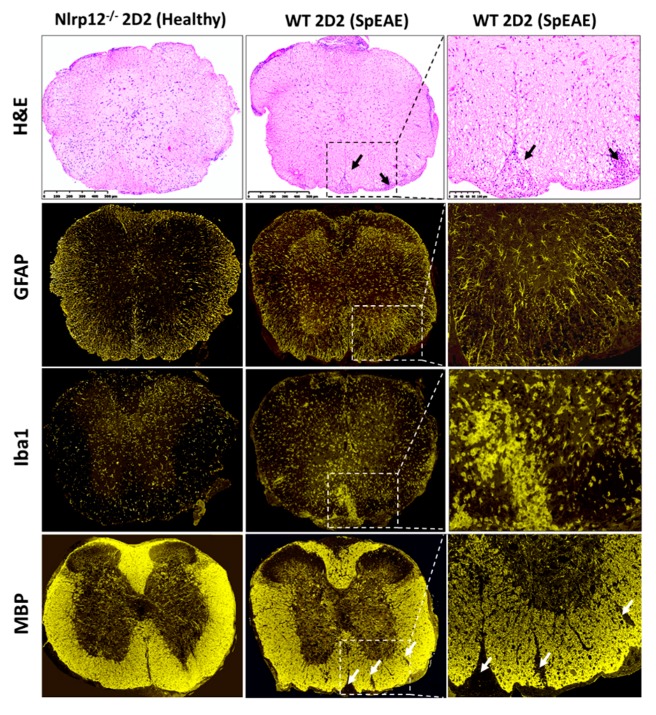
SpEAE in WT 2D2 mice is associated with spinal cord inflammation and demyelination. Representative histopathological examination of the spinal cords from healthy *Nlrp12^−/−^* 2D2 mice and spEAE WT 2D2 mice. H&E staining reveals an extensive mononuclear cell infiltration to the spinal cord of spEAE mice (shown by black arrows). Immunofluorescent staining of spinal cords from WT 2D2 spEAE shows an increased expression of GFAP (astrocyte marker) and Iba1 (macrophage/microglia marker) mice compared to *Nlrp12^−/−^* 2D2 healthy mice. Focal demyelinating lesions (shown by white arrows) are observed in spEAE spinal cords using MBP staining. Similar histopathological features to healthy *Nlrp12^−/−^* 2D2 mice were found in healthy WT 2D2 mice (images not shown).

**Figure 9:**
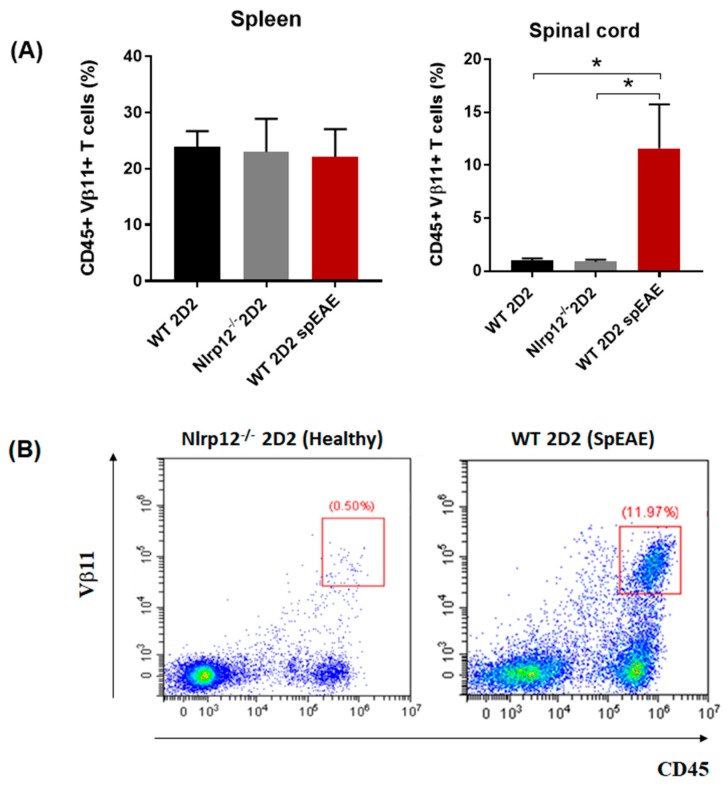
Myelin-specific T cells infiltrate to the spinal cord of spEAE mice. (**A**) The percentage of T cells expressing myelin-specific transgenic TCR (Vβ11^+^) were comparable in the spleen of healthy and spEAE mice, however, the percentage of Vβ11^+^ T cells were increased in the spinal cord of spEAE mice compared to WT healthy and *Nlrp12^−/−^* healthy mice (*n* = 6), * *p* < 0.05; (**B**) Representative flow cytometry plots of CD45^+^ Vβ11^+^ T cells in the spinal cord of *Nlrp12^−/−^* 2D2 healthy and WT 2D2 spEAE mice.

Given the anti-inflammatory role of *Nlrp12* in EAE [19,20], we investigated whether *Nlrp12^−/−^ 2D2* mice would develop spEAE. As expected, 6% of WT 2D2 mice developed EAE spontaneously (Table 1). However, surprisingly, none of *Nlrp12^−/−^* 2D2 mice developed spEAE (Table 1). Pathological examination of WT 2D2 spEAE mice revealed marked inflammation associated with infiltration of mononuclear cells to the spinal cord and increased expression of microglia and astrocyte markers (Iba1 and GFAP respectively) (Figure 8). The inflammation was associated with demyelination, as shown in by immunofluorescence staining of myelin basic protein (MBP) (Figure 8). Using flow cytometry, we found a similar percentage of myelin-specific T cells (Vβ11^+^) in the spleens of *Nlrp12^−/−^* 2D2 mice and WT 2D2 mice (Figure 9). However, a high percentage of leukocytes (CD45^high^) including Vβ11^+^ T cells infiltrated to the spinal cord of WT 2D2 spEAE compared to healthy mice (Figure 9).

## 4. Discussion

Early reports showed the expression and anti-inflammatory function of *Nlrp12* in innate immune cells of myeloid origin such as DC and macrophages [18,26]. However, a very recent report by Lukens et al. revealed the expression of *Nlrp12* in T cells [20]. The current study aimed to investigate the immunoregulatory function of *Nlrp12* in T cell-mediated immune response in EAE. Our results suggest that *Nlrp12* plays pivotal role in Th1/Th2 balance by inhibiting Th1 peripheral responses in the favor of Th2. We demonstrated that in - lymph nodes of *Nlrp12^−/−^* mice, Th1 to Th2 ratio is increased compared to WT mice. This shift, in part, can be explained by significant increases in the production of IFNγ by *Nlrp12^−/−^* T cells. Interestingly, *Nlrp12* does not play a role in the differentiation of naïve T cells but upregulates IL-2 production and proliferation of CD4^+^ T cells. The effect of *Nlrp12* is associated with its increased expression and inhibition of major molecular pathways including Akt and NF-κB in activated T cells. 

Previously, we published that *Nlrp12^−/−^* mice develop more severe form of EAE than WT mice, which is associated with exacerbated spinal cord inflammation and increased activation of microglia in *Nlrp12^−/−^* mice compared to WT mice [19]. In the present study, we found an increased Th1 dominant response in lymph nodes of *Nlrp12^−/−^* EAE mice, suggesting that *Nlrp12* suppresses Th1 activation in the periphery. Interestingly, we did not find any change in the levels of IFNγ and IL-4 in the CNS from *Nlrp12^−/−^* EAE mice compared to WT EAE mice. However, the IFNγ/IL-4 ratio significantly decreased in the spinal cord of *Nlrp12^−/−^* EAE mice compared to WT EAE mice, which is consistent with Lukens et al. observation of enhanced Th2 response and increased IL-4 production in the CNS of *Nlrp12^−/−^* EAE mice [20]. In contrast to Lukens’ study where *Nlrp12^−/−^* mice developed atypical EAE signs, we found severe classical EAE signs in *Nlrp12^−/−^* mice compared to WT mice [19]. Several plausible explanations of these discrepancies were proposed in our recent review [9]. One possibility might be related to the difference in MOG-adjuvant immunization protocols between various labs. In our report, WT and *Nlrp12^−/−^* animals were immunized with a total dose of 200 µg MOG [19], which is two-fold higher than the immunization dose used by Lukens et al. [20]. Interestingly, a recent study showed that immunization with low or high MOG concentration can modify the patterns of inflammatory cytokines [27]. Their study demonstrates that anti-inflammatory cytokines such as IL-10 and TGFβ significantly increase in the CNS of EAE animal immunized with 100 µg MOG compared to 300 µg MOG immunization. Therefore, it is possible that the severe EAE signs in *Nlrp12^−/−^* mice in our study were driven by lower levels of IL-10 and TGFβ anti-inflammatory cytokines or by higher levels of other inflammatory cytokines such as Granulocyte-macrophage colony-stimulating factor (GM-CSF) in the CNS [28,29]. Another possible explanation for observing different EAE profiles is the difference in the environmental conditions and different knockout strategies. It was shown that some C57BL/6 colonies have acquired a missense mutation in the *Nlrp12* gene that can affect neutrophil responses [30]. In another study, genetic ablation of *Nlrp12* was found to cause significant changes in microbiota [17]. Nevertheless, these variabilities highlight the complex immunoregulatory nature of *Nlrp12* that warrants further investigation. 

Given the important role of T cells in the pathogenesis of EAE, we further investigated whether *Nlrp12* controls T cell proliferation and activation in a T cell-intrinsic manner. We found a significant increase of IFNγ and IL-4 levels in the supernatant of *Nlrp12^−/−^* compared to WT T cells. However, when we measured the levels of intracellular cytokines by flow cytometry, higher percentage of CD4^+^ IFNγ^+^ T cells were found in *Nlrp12^−/−^* group, while the percentages of CD4^+^ IL-4^+^ T cells or CD4^+^ IL-17^+^ T cells did not change between both groups. We addressed this discrepancy with Ki67 staining and our flow cytometry results revealed that activated *Nlrp12^-/-^* T cells proliferate significantly more than WT T cells, which explains why we found increased production of both IFNγ and IL-4 in the supernatant of activated *Nlrp12^−/−^* T cells. Consistent with our findings, Lukens et al., observed the higher expression of activation markers, enhanced proliferation and elevated secretion of Th1/Th2/Th17 cytokines by *Nlrp12^−/−^* T cells compared to WT T cells in vitro [20]. Collectively, these results show that *Nlrp12* inhibits the activation of inflammatory T cell subsets including Th1 and Th17.

In the presence of polarizing cytokines, we found no difference between *Nlrp12^−/−^* or WT T cells in the expression of Th1- or Th17- associated molecules, suggesting that *Nlrp12* does not affect the differentiation of naïve T cells to Th1 or Th17. In a recent study by Cai et al., purified CD4^+^ T cells from *Nlrp12^−/−^* or WT mice were activated with anti-CD3 and polarizing cytokines for 6 days. They showed that the differentiation of *Nlrp12^−/−^* T cells to Th1 or Th17 cells were significantly lower than WT T cells. However, no difference was found between WT and *Nlrp12^−/−^* T cells in Th2 differentiation [31]. Taken together, it appears that *Nlrp12^−/−^* T cells respond differently to the environmental stimuli, depending on the type of activating signals, incubation period and polarizing conditions.

Our results, in agreement with Lukens’ study [20], showed that *Nlrp12* inhibits T cell activation and proliferation. We found that the expression of *Nlrp12* was significantly increased in T cells following activation by anti-CD3 or anti-CD3/CD28 antibodies. The increased level of *Nlrp12* expression remained high even after 48 h stimulation of T cells, suggesting that *Nlrp12* modulates T cell signaling pathways upon TCR stimulation. 

**Figure 10:**
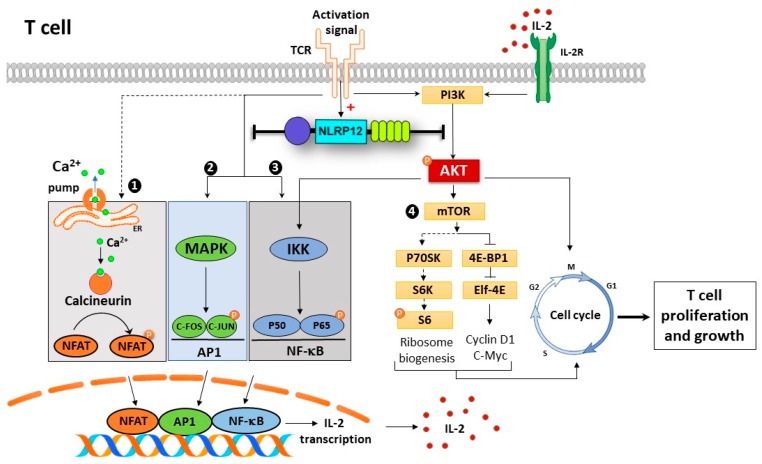
Possible mechanism of action of Nlrp12 in regulating early TCR signaling pathways, T cell activation and proliferation. Activation signals from TCR activates multiple downstream signaling pathways in T cells, presented as (1) NFAT, (2) NF-κB, (3) MAPK and (4) mTOR pathways. These pathways lead to nuclear translocation of NFAT, NF-κB and AP1 transcription factors and IL-2 transcription. IL-2 binds to its receptor on the cell surface and initiates mTOR pathway. TCR activating signals induce the expression of *Nlrp12* that consequently inhibits NF-κB and MAPK signaling pathways, which suppress IL-2 production. Upstream of NF-κB pathway, *Nlrp12* inhibits Akt phosphorylation that controls NF-κB signaling on one side and mTOR activity and cell cycle progression on the other side. Taken together, *Nlrp12* has a broad range of regulatory activity that controls hyper-proliferation and activation of T cells. The pathways shown in dashed lines are not affected by *Nlrp12* including NFAT signaling and mTOR/S6 phosphorylation.

Since multiple signaling pathways are involved in T cell activation and proliferation, we hypothesized that signaling pathways were inhibited by *Nlrp12* in activated T cells. It is well-established that TCR activation triggers calcineurin (a Ca^2+^/calmodulin-dependent phosphatase), MAPK and NF-κB signaling pathways, leading to the activation of the nuclear factor of activated T cell (NFAT), AP1 and NF-κB transcription factors that initiate IL-2 transcription [22]. In this regard, we evaluated the phosphorylation of p65 (NF-κB subunit) and Akt in activated CD4^+^ T cells. Western blot results demonstrated a significant increase in the phosphorylation of p65 in activated *Nlrp12^−/−^* T cells compared to WT T cells, highlighting the inhibitory effect of *Nlrp12* on NF-κB signaling pathway (Figure 10). These results are in agreement with previous studies that show *Nlrp12* suppresses canonical and non-canonical NF-κB pathways [16,20,32,33]. 

The NF-κB signaling cascade interacts with several parallel pathways including the signaling cascades initiated by phosphatidylinositol 3-kinase (PI3K) and Akt. We found a significant increase of Akt(Ser 473) phosphorylation in *Nlrp12^−/−^* T cells compared to WT T cells. These results are supported by a study that demonstrates *Nlrp12* negatively regulates Akt signaling pathway in affected tumor tissues in colitis model [16]. Interestingly, Akt acts upstream of NF-κB, where the activation of PI3K/Akt signaling pathway leads to phosphorylation and degradation of IκB protein, resulting in nuclear translocation and transcriptional activation of NF-κB [34]. Taken together, our results demonstrate that *Nlrp12* inhibits Akt signaling pathway, which, subsequently, affects downstream pathways including NF-κB pathway. Moreover, phosphorylated Akt blocks the activity of two G1-checkpoint inhibitors, p21 and p27, and promotes cell cycle progression [35]. Collectively, our results suggest that *Nlrp12* controls T cell proliferation and cytokine production by inhibiting the activation of Akt, that, in turn, affects several downstream effectors (Figure 10).

One of the signaling pathways downstream of Akt phosphorylation is mTOR pathway, which induces protein synthesis and cell growth by regulating ribosomal p70S6 kinase 1 (S6K1) and eukaryotic translation factor 4E-binding protein 1 (4EBP1). S6K1 phosphorylates and activates S6, a ribosomal subunit involved in initiating protein synthesis machinery. Using western blotting and flow cytometry, we found no difference between *Nlrp12^−/−^* T cells and WT T cells in the level of S6 phosphorylation. Therefore, it is possible that *Nlrp12* regulates protein synthesis and cell growth via modulating the activity of 4EBP1 (Figure 10). Further investigation is warranted to uncover the regulatory mechanism of *Nlrp12* on mTOR signaling pathway.

The results of this study and previous publications suggest that *Nlrp12* inhibits NF-κB and MAPK signaling pathways in activated T cells [20,36]. The transcription of IL-2 gene is regulated by NF-κB, MAPK and Ca^2+^/calmodulin-dependent pathways [37,38]. Accordingly, we hypothesized that *Nlrp12* inhibits IL-2 production by activated CD4^+^ T cells. Flow cytometry data revealed a higher percentage of CD4^+^ IL-2^+^ T cells in activated *Nlrp12^−/−^* CD4^+^ cells compared to WT CD4^+^ T cells, suggesting that *Nlrp12* suppresses IL-2 production by activated T cells. Incubation with cyclosporin inhibited IL-2 production by both *Nlrp12^−/−^* and WT T cells, indicating that lack of *Nlrp12* does not affect Ca^2+^/calmodulin signaling pathway in T cells. 

**Figure 11:**
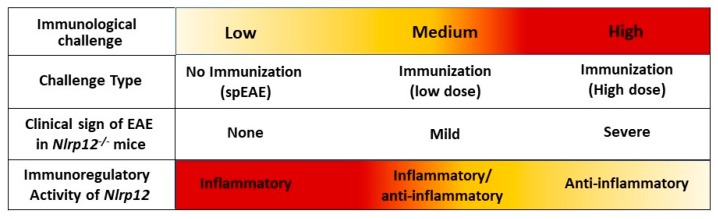
Dual immunoregulatory function of *Nlrp12* in EAE. The severity of immunological challenge, ranging from low to high, can influence the regulatory function of *Nlrp12* in EAE. In this model, when there is no MOG/CFA immunization, myelin-specific T cells face a low level of challenge. In this case, *Nlrp12* plays an inflammatory role, which explains why WT 2D2 mice developed spEAE and *Nlrp12^−/−^* 2D2 mice were resistant to spEAE. On the other hand, with MOG/CFA immunization, T cells encounter immunological challenge that may vary from medium to high levels. In mild challenge, perhaps with lower dose of MOG-CFA immunization, *Nlrp12* is a bifunctional molecule, acting as an inflammatory or anti-inflammatory molecule. In this condition, EAE mice developed mild EAE signs, mostly atypical symptoms such as ataxia [20]. When MOG/CFA immunization provides high levels of immunological challenge, *Nlrp12* acts as an anti-inflammatory molecule, inhibiting severe signs of classical EAE [19]. Given the fact that *Nlrp12* ligand is still unknown, what exactly tunes *Nlrp12* response as a pro–inflammatory or anti-inflammatory molecules, needs further investigation.

Our in vitro findings, together with results obtained from in vivo EAE model, support the idea that *Nlrp12* inhibits T cell responses. In 2D2 mice, due to the presence of many MOG-reactive T cells, about 4–14% of mice developed spEAE [39,40]. In 2D2 mice, the percentage of Vβ11^+^ T cells in the spleen were the same between healthy and spEAE animals, showing that our affected and non-affected animals had similar percentage of MOG-reactive T cells. However, a high percentage of MOG-reactive T cells infiltrated to the spinal cord of WT 2D2 spEAE mice, which confirms the presence of autoreactive T cells in inflamed spinal cord. The healthy *Nlrp12^−/−^* 2D2 animals have only a few Vβ11^+^ T cells in the spinal cord and do not contain pathology. The fact that none of *Nlrp12^−/−^* 2D2 mice develop disease suggests that *Nlrp12* does not inhibit the development of spEAE and even can serve as contributing factor in the pathology of EAE. Due to slow rate of breeding of *Nlrp12^−/−^* 2D2 mice (unpublished observation), these conclusions are based on the observation of thirty *Nlrp12^−/−^* 2D2 mice. Interestingly, previous studies report that in induced EAE, *Nlrp12* can prevent [19] or promote [20] CNS inflammation. To explain the observed controversy, we propose a dual immunoregulatory function for *Nlrp12,* in which *Nlrp12* can act as an inflammatory or anti-inflammatory molecule, depending on the type and severity of immunological challenge. The hypothetical model of *Nlrp12* immunoregulation is shown in Figure 11. 

The bifunctional nature of *Nlrp12* has been previously reported in several studies [16,19,20,31,36,41,42]. Early in vitro studies suggest that *Nlrp12* is an inflammatory NLR that interacts with ASC to form inflammasome [43]. Recent reports also support this idea and show that *Nlrp12* activates inflammasome in *Yersinia Pestis* and *Plasmodium* infections [42,44]. Moreover, several behavioral outcomes are similar between *ASC^−/−^* and *Nlrp12^−/−^* mice [45]. On the other hand, there are studies that classify *Nlrp12* as an anti-inflammatory molecule and the inhibitor of NF-κB signaling pathway [16,18,32,46]. In this regard, *Nlrp12^−/−^* mice were shown to be highly susceptible to inflammatory diseases of intestine such as experimental colitis and colon cancer [16,18]. Taken together, these findings support the dual immunoregulatory nature of *Nlrp12*, that may vary in a cell-specific or stimulus-specific manner. 

In conclusion, the present study provides new insight into the immunoregulatory role of *Nlrp12* in T cell-mediated CNS inflammation, triggered by different modes of immunological challenges (induced EAE and spEAE). We demonstrated for the first time that *Nlrp12* inhibits early TCR signaling pathways. In this way, *Nlrp12* plays critical roles in balancing T cell response to control overt activation and maintain cellular homeostasis. Indeed, the fine-tuned triggering of *Nlrp12* by different immunological challenges determine whether the outcome of the challenge would be an inflammatory or anti-inflammatory response. Factors determining the immunoregulatory function of *Nlrp12* remain to be determined.

## Figures and Tables

**Table 1:** *Nlrp12* does not prevent the development of spEAE. None of *Nlrp12^−/−^* 2D2 mice developed spEAE, while 6 WT 2D2 mice out of 101 developed spEAE, manifested with ascending paralysis. *Nlrp12^−/−^* 2D2 mice were monitored for 16 weeks and no sign of classical EAE was observed.

Genotype	Total (*n*)	SpEAE (%)	Age of Onset (Weeks)	EAE Score
WT 2D2	101	6	10.1 ± 4.7	3.6 ± 0.5
*Nlrp12^−/−^* 2D2	30	0	-	-

## Supplementary Materials

The following are available online at http://www.mdpi.com/2073-4409/7/9/119/s1, Figure S1: NLRP12 genotyping of a *Nlrp12^-/-^* 2D2 mouse and a WT 2D2 mouse. DNA was obtained from ear samples, Figure S2: Clinical score of induced EAE in *Nlrp12^-/-^* mice compared to WT mice.
