# Supplementary material for: The Dual Immunoregulatory function of Nlrp12 in T Cell-Mediated Immune Response: Lessons from Experimental Autoimmune Encephalomyelitis
Source: Cells. 2018 Aug 27;7(9):119. doi: 10.3390/cells7090119 (PMC6162721; doi:10.3390/cells7090119)
Supplement: Supplementary file 1 [file cells-07-00119-s001.pdf]

Supplementary Information:

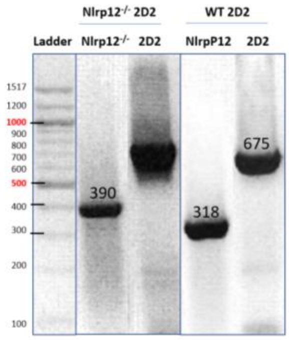

Figure S1. NLRP12 genotyping of a *Nlrp12*$^{-/-}$ 2D2 mouse and a WT 2D2 mouse. DNA was obtained from ear samples.

Genotyping of *Nlrp12*$^{-/-}$ 2D2 mice

Genotyping of *Nlrp12*$^{-/-}$ mice was performed as previously described[47]. Molecular weights for *Nlrp12*$^{-/-}$ and WT bands are 390 and 318 base pairs (bp) respectively. Genotyping of 2D2 mice was performed based on Jackson laboratory protocol (Tg(Tcra2D2)1Kuch). Molecular weight for 2D2 band is 675 bp.

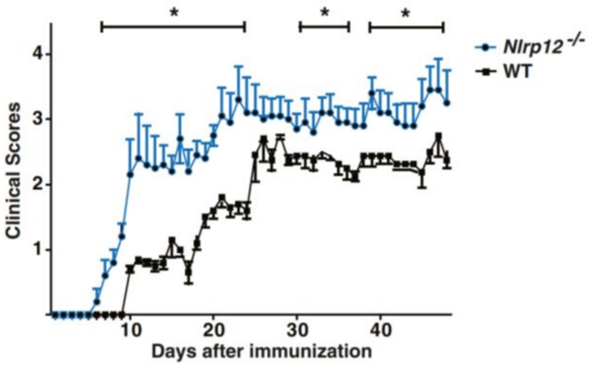

Figure S2. Clinical score of induced EAE in *Nlrp12*$^{-/-}$ mice compared to WT mice, published by Gharagozloo et al. in J Neuroinflammation, 2015. *Nlrp12*$^{-/-}$ mice developed earlier and exacerbated EAE compared to WT mice after MOG-CFA immunization. Animals were scored daily based on the following scale: 0, no sign of disease; 1, limp tail or weakness in limbs; 2, limp tail and weakness in limbs; 3, partial limb paralysis; and 4, complete limb paralysis. Statistical analysis was done by Kruskal-Wallis one-way ANOVA test followed by Bonferroni multiple comparison test. $n = 7$, * $p < 0.05$.
